# Liposomes for Magnetic Resonance Image-Guided Drug Delivery; Lipid Chain Length Affects Drug Release and MRI Relaxivity

**DOI:** 10.3390/molecules30081729

**Published:** 2025-04-11

**Authors:** Paul Cressey, Jacob C. Wilson, Maral Amrahli, Maya Thanou

**Affiliations:** School of Cancer and Pharmaceutical Sciences, Institute of Pharmaceutical Science, King’s College London, London SE1 9NH, UK; paul.cressey@kcl.ac.uk (P.C.); jacob.c.wilson@kcl.ac.uk (J.C.W.); maral.amrahli@kcl.ac.uk (M.A.)

**Keywords:** drug release, liposome, magnetic resonance imaging (MRI), thermosensitive

## Abstract

Image-guided drug delivery is a method for tracking drug carriers for activation in specific lesions in the body. Image guidance uses the labelling of the drug or carrier and a clinically approved imaging modality. MRI (magnetic resonance image)-guided drug delivery has been considered for focused ultrasound tumour-targeted drug release. Liposomes are labelled for MRI tracking and the confirmation of drug delivery. In this study, we prepared two lipids conjugated to Gd-DOTA that confer MR imaging properties. Two lipid conjugates to DOTA, a C_18_ (**LCA-1**) and a C_16_ (**LCA-2**), were synthesised. The lipids were combined at different ratios within the lipid mix, and we investigated their effects on the liposome’s T_m_ using DSC (differential scanning calorimetry) and on relaxivity using NMR. The results show that when different combinations of **LCA-1** and **LCA-2** were introduced into the liposomes, their ratio affected both thermal drug release and relaxivity. As these lipids are part of the liposomal membrane, they confer tracking ability, and their effect on relaxivity due to thermal release could enable the confirmation of liposomal drug release using MRI at clinically relevant magnetic field strengths.

## 1. Introduction

The ever-increasing prevalence of cancer is still at the forefront of medical research due to multiple drawbacks in treatment options [1]. One of these key limitations is the poor delivery of drugs to the tumour site, which often results in harmful and unpleasant off-target effects [2,3,4]. Drug delivery vehicles work to protect the agent from physiological degradation and to extend the agent’s half-life, as well as potentially enabling selective release at the desired site to reduce off-target effects [5,6].

The introduction of purpose-made lipids into the liposomal formulation is seen as a way to attain specific properties (e.g., imaging) and also adjust the drug release profile. For example, introducing cholesterol could alter the rigidity of the liposomal membrane [7]. Alternatively, the shortening/lengthening of the lipid alkyl chain length and the saturation of the lipid chain can be used. Lengths of C_4_ to C_18_ have been investigated and have been shown to have a significant effect on the rigidity of the lipid membrane, with rigidity shown to increase with the lipid chain length. [8,9] With the alternative adjustment of the saturation of the lipid chain, the introduction of unsaturated π bonds influences the stability and rigidity of the liposome; for example, the use of unsaturated DOPC versus saturated DPPC alongside cyclodextrins has been shown to add stability but results in slower drug release from liposomal formulations [10,11].

External sources can be used to stimulate targeted drug delivery, including light, heat, magnetic fields, and ultrasound [12,13]. In particular, high-intensity ultrasound has been leveraged to initiate the release of liposomal payloads and improve drug efficacy in preclinical models by inducing highly localised hyperthermia [14,15,16].

The use of magnetic resonance imaging (MRI) via the introduction of contrast agents into the liposomal formulation has been suggested for monitoring stimulus-induced drug delivery. An example of this is gadolinium-loaded liposomes, which were used to image solid pancreatic tumours with mild hyperthermic environments used to release the contrast agent at the tumour site [17]. However, issues with this technique have been observed due to the small imaging window once the encapsulated agent is released, requiring longer infusion times or specialised MRI sequences, as well as the rapid diffusion of the contrast agents being released, as seen in organs such as the brain [18].

Alternatively, the MR contrast agent can be embedded in or bound to the lipidic membrane to inherently track the liposomes themselves. This would reduce the diffusion of the contrast agent, allowing longer and easier imaging windows to track the drug delivery vehicles. Examples of this include the use of a Gd-DTPA lipid for in vitro relaxometry and a Gd-DOTA-DSA derivative used for in vivo ovarian cancer MR imaging [19,20]. The ability to image liposome delivery is a key attribute, as it will enable the visualisation and tracking of drug delivery in real-time, furthering their potential as ‘theranostic’ platforms [21,22].

However, the ability to finetune liposomal drug release while maintaining the ability to monitor drug delivery has not been explored. Here, we investigate the effect of the lipid chain length on the drug release and MRI contrast of imageable thermosensitive liposomes. The lipid MRI contrast agents synthesised in this study were **LCA-1** with a C_18_ chain length and **LCA-2** with a C_16_ chain length coupled to a Gd-DOTA headgroup (1,4,7,10-Tetraazacyclododecane-1,4,7,10-tetraacetic acid, DOTA). These MR agents were synthesised using a modified method published by Kamaly et al. [23]. The thermosensitive liposomes were formulated with different ratios of **LCA-1** and **LCA-2** and then investigated to determine how this variation in chain length affects their physicochemical characteristics, including size, surface charge, gadolinium concentration, thermal membrane transitions, topotecan drug release properties, and relaxometry.

## 2. Results and Discussion

### 2.1. Synthesis of **LCA-1** and **LCA-2**

**Scheme 1:**
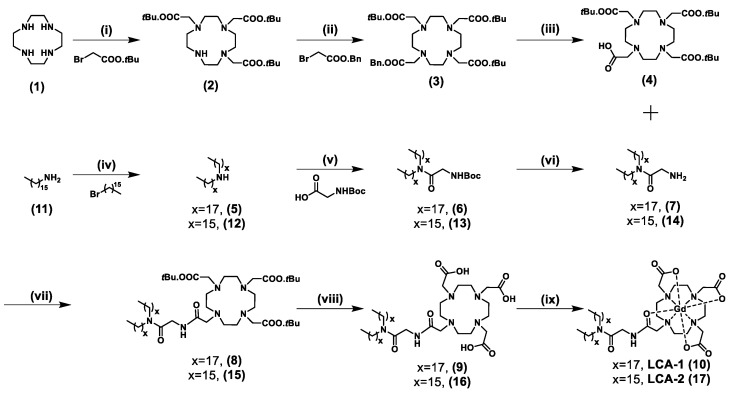
Synthesis of **LCA-1** and **LCA-2**. Reagents and conditions: (**i**) **1**, BrCH_2_COO*t*Bu, NaHCO_3_. CH_3_CN, RT, **2** = 66%. (**ii**) **2**, K_2_CO_3_, CH_3_CN, 70 °C, **3** = 72%. (**iii**) **3**, H_2(g)_, Pd(OH)_2_/C, MeOH, RT, **4** = 94%. (**iv**) **11**, Br(CH_2_)_15_CH_3_, K_2_CO_3_, MeCN, **12** = 63%. (**v**) **5/12**, HBTU, DIPEA, OH.Glycine.BOC, CH_2_Cl_2_, RT, **6** = 90%, **13** = 75%. (**vi**) **6/13**, TFA: DCM (3:7), RT, **7** = 97%, **14** = 97%. (**vii**) **4**, **7/14**, HBTU, DIPEA, CH_2_Cl_2_, RT, **8** = 45%, **15** = 70%. (**viii**) **8/15**, HCl:dioxane (4M) 60 °C, **9** = 89%, **16** = 75%. (**ix**) **9/16**, GdCl_3_^.^6H_2_O, H_2_O, pH 6, 100 °C, **LCA-1** (**10**) = 70%, **LCA-2** (**17**) = 80%.

The method used to synthesise the lipid contrast agents **LCA-1** and **LCA-2** was a modification of the synthetic route previously reported by Kamaly et al. [20] The lipid comprises a Gd-DOTA-mono amide head group conjugated to a C_18_ or C_16_ alkyl chain moiety to anchor the conjugate to the liposome lipid membrane. Conjugation was achieved using biodegradable amide groups. The novelty of the lipid arises from the shorter C_16_ alkyl chain lipid tail (Scheme 1). These shorter-lipid-tail analogues were synthesised as a way of modulating the phase transition T_m_ of thermosensitive liposomes while maintaining MR contrast.

The synthesis of **LCA-1** and **LCA-2** was developed as a convergent synthesis in which the headgroup (OH-(*t*Bu)_3_-DOTA) was synthesised separately from the alkyl lipid tail. The synthesis of the OH-(*t*Bu)_3_-DOTA headgroup (**4**) began with cyclen (**1**), which was alkylated with bromide to give (*t*Bu)_3_-DO3A (**2**), indicated via the presence of two BOC peaks (18H and 9H) on the ^1^H-NMR spectrum. This was further alkylated with bromide to give **3**, a mono-benzyl-protected, triple-tert-butyl-protected DOTA (Bn-(*t*Bu)_3_-DOTA), with the presence of a 5H benzyl aromatic multiplet on the ^1^H-NMR spectrum confirming a successful reaction. Removal of the benzyl was performed via catalytic hydrogenation using 10% Pd(OH)_2_/C in methanol to yield the monocarboxylate-(*t*Bu)_3_-DOTA (**4**), indicated on the ^1^H-NMR spectrum with the disappearance of the 5H benzyl aromatic multiplet.

The synthesis of the alkyl lipid tail was achieved via amide coupling of the dialkyl amines **5** and **12** (purchased commercially) and OH.glycine.BOC using HBTU and DIPEA as carboxylic acid activators to yield **6** and **13**. Successful coupling was confirmed by the presence of a 9H BOC peak in the ^1^H-NMR spectrum. The BOC-protected amine group was deprotected using 30% TFA in DCM to yield **7** and **14** as a TFA salt, with the disappearance of the 9H BOC peak on the ^1^H-NMR spectrum indicating successful deprotection. An additional step in the synthesis of the lipid tail for the C_16_ tail-length lipid conjugate was required, as the dialkylated amine was not commercially available. Therefore, the di hexadecyl amine was synthesised by mono-alkylation of **11** with bromide to give **12**, resulting in a mass increase on ESI-MS of 225.3 a.m.u. The building blocks **4** and **7/14** were amide-coupled using HBTU and DIPEA to form **8** and **15**, using ^1^H-NMR spectroscopy via the presence of both the 28H cyclic headgroup multiplet and the 52H (C_16_) or 60H (C_18_) alkyl lipidic singlet to determine successful coupling. Deprotection of the *t*ert-butyl groups was carried out using HCl: Dioxane (4 M) at reflux to yield the deprotected DOTA.lipid conjugates **9** and **16**, confirmed by ^1^H-NMR spectroscopy with the disappearance of the 27H BOC doublet as well as ESI-MS with the reduction *m*/*z* to 987.7 ([M + Na^+^]^+^) and 909.8 ([M + H^+^]^+^) a.m.u. for conjugates **9** and **16**, respectively. The chelation of gadolinium into the deprotected DOTA head group was achieved by the addition of Gd^3+^ (GdCl_3_^.^6H_2_O) at pH 5 under reflux to yield the final products **LCA-1** (**10**) and **LCA-2** (**17**), which were confirmed using ESI-MS ([**LCA-1** + H^+^]^+^ = 1120.1 a.m.u. and [**LCA-2** + H**^+^**]^+^ = 1064.2 a.m.u.).

### 2.2. Thermosensitive Liposome Preparation and Characterisation

**Figure 1:**
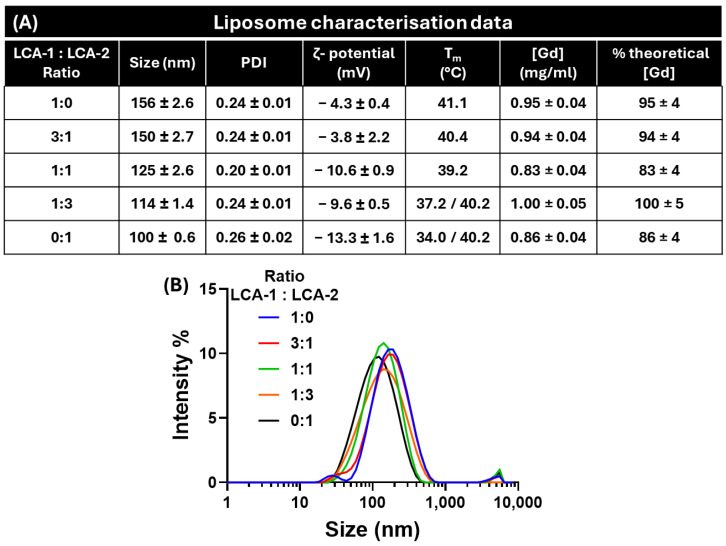
**Liposomal nanoparticle characterisation**; (**A**) **Representative colloidal data** for various ratios of **LCA-1**:**LCA-2**, including hydrodynamic size, PDI, polydispersity index, ζ-potential, transition temperature (T_m_), and [Gd] concentration. (**B**) **Dynamic light scattering curves** of the five ratios of contrast lipids: **LCA-1**:**LCA-2**; 1:0 (blue), 3:1 (red), 1:1 (green), 1:3 (orange), and 0:1 (black). All values are mean average ± 1 SD; n = 3.

Liposome preparation was carried out using a freeze–thaw method, following reported protocols [24]. Briefly, a lipid film of 45 mg was prepared containing the following lipids: DPPC: MSPC: DSPE.PEG_2000_OMe: **LCA-1/2** (55.3: 9.7: 5: 30 mol%). It was dried overnight under vacuum. **LCA-1** and **LCA-2** were added at predefined ratios (**LCA-1**/**LCA-2**; Figure 1A) to total incorporation of DOTA.lipid of 30 mol%. The lipid film was hydrated in a topotecan (1 mg/mL) buffer of 300 mM NH_4_SO_4_, pH 3 (1.5 mL, 30 mg/mL lipid). The film was then fragmented by alternately plunging into liquid nitrogen and then hot water to form multi-lamellar vesicles. The resulting suspension was sonicated at 60 °C to form a homogeneous opalescent solution of unilamellar liposomes. After the formation of the liposomes, PD10 size exclusion column chromatography was carried out to remove unencapsulated free drug and to exchange the external buffer for 20 mM HEPES, 5% glucose, pH 7.4.

After purification, the liposomes were characterised in terms of critical parameters (Figure 1A). The first is their hydrodynamic size and polydispersity index (PDI) via dynamic light scattering (DLS, Figure 1B). All liposomes produced formed stable species ranging between 100 and 160 nm, with a narrow polydispersity index (<0.3 PDI). For liposomes with a 1:0 ratio of **LCA-1**:**LCA-2**, their size was 156 ± 2.6 nm. As the ratio of **LCA-2** increased, liposomes showed a decrease in size, ending with a size of 100 ± 0.6 nm for the 0:1 ratio of **LCA-1**:**LCA-2**. This was due to the decreasing length of the alkyl ‘tails’ shifting the lipid shape towards a more inverted conical shape. Therefore, it is possible that the higher proportion of inverted conical-shaped lipids (in this instance, **LCA-2**) within the liposome results in smaller liposomes as the preferred supramolecular assembly of the formulation shifts away from the bilayer towards a more micellar assembly [25,26].

The ζ-potential of the nanoparticles is also important, with this value quantifying the surface charge of the liposomes that will affect their fate in vivo (biodistribution). All formulations were classified as almost neutral, with ζ-potentials between −3 and −13 mV.

The gadolinium content of the liposomes was determined via ICP-MS using gallium (10 mg/L) as an internal standard. Between 0.8 and 1.0 mg/mL of gadolinium was embedded in each liposomal formulation, resulting in encapsulation efficiencies of over 83%. This was deemed optimal due to potential losses in the PD10 purification process required to remove unencapsulated Topotecan from the liposomal formulation.

### 2.3. Lipid Phase-Transition Temperature Analysis of **LCA-1**:**LCA-2** Liposomes

To determine the effect of the **LCA-1**:**LCA-2** ratio on the phase transition properties of the liposomes, DSC (differential scanning calorimetry) studies were performed. Liposomes were diluted to (1 mg/mL) and underwent three rounds of sequential heating/cooling (25–70 °C at 1 °C/min) to determine the T_m_ (transition temperature).

**Figure 2:**
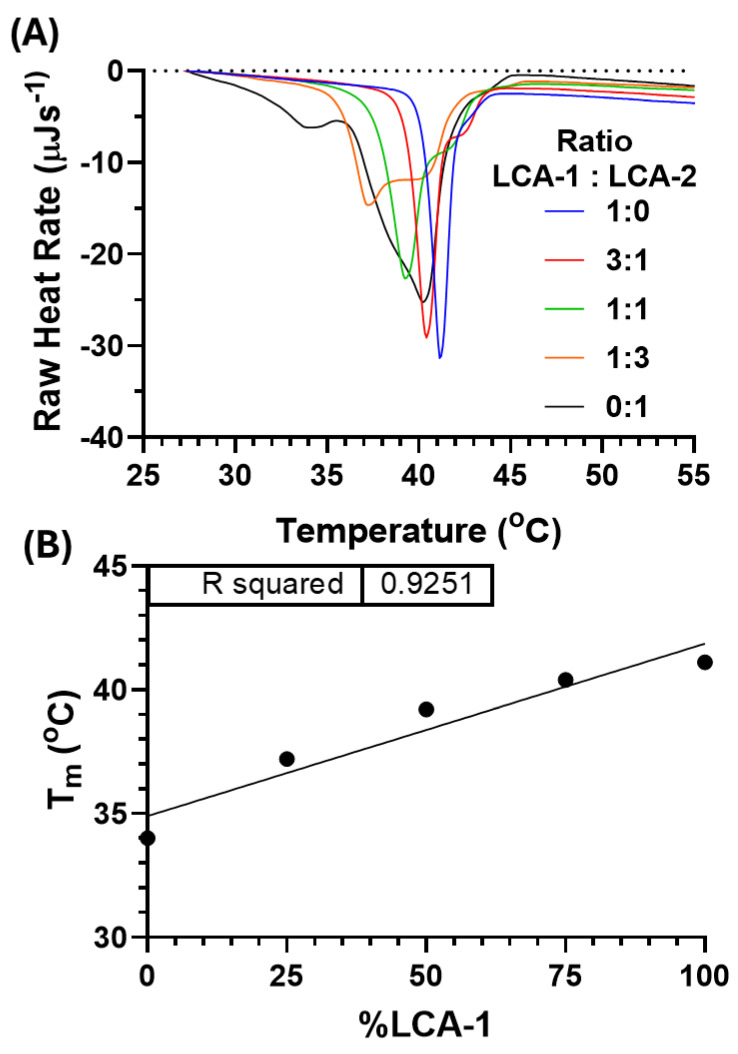
(**A**) **Differential scanning calorimetry (DSC) thermographs** for the five ratios of contrast lipids, **LCA-1**:**LCA-2**: 1:0 (blue), 3:1 (red), 1:1 (green), 1:3 (orange), and 0:1 (black). DSC measurement included ×3 heating/cooling cycles over 25–70 °C at 1 °C/min. Transition temperature (T_m_) for each cooling thermograph for **LCA-1**:**LCA-2**: 1:0 (41.1 °C, blue), 3:1 (40.4 °C, red), 1:1 (39.2 °C, green), 1:3 (37.2 °C, orange), and 0:1 (34.0 °C, black). (**B**) **Linear correlation between T_m_ and LCA substitution** (R^2^ = 0.9251). The dotted line indicates zero raw heat rate.

Representative cooling curves were selected and normalised for analysis (Figure 2A). The ratio of **LCA-1**:**LCA-2** had a significant effect on the T_m_ of the formulations, with a shift from 41.1 °C to 40.4, 39.2, 37.2, and 34.0 °C for **LCA-1**:**LCA-2**; 1:0, 3:1, 1:1, 3:1, and 0:1, respectively. The trend observed for the formulations shows that by increasing the proportion of **LCA-1** in the formulation, the thermal transition temperature increases. This change in T_m_ ranges from activatable below body temperature to mild hyperthermia. As expected, there is a strong correlation between the **LCA-1**:**LCA-2** ratio and the T_m_, with an R^2^ value of 0.9251 (Figure 2B). A further effect was observed when the proportion of **LCA-2** was above **LCA-1**; the T_m_ peak split into two peaks at 37.2 °C/40.2 °C and 34.0 °C/40.2 °C for the 1:3 and 0:1 ratios of **LCA-1**:**LCA-2**. This peak-splitting within the DSC could be due to the non-homogeneous lipid mixing between the varied-chain length lipids within the bilayer, resulting in two separate T_m_ peaks. A similar effect has been seen previously, where this peak-splitting phenomenon was observed when numerous lipids of varied lengths were introduced into liposomal formulations [27,28].

### 2.4. Topotecan Release Profile with Varied **LCA-1**:**LCA-2** Ratios

**Figure 3:**
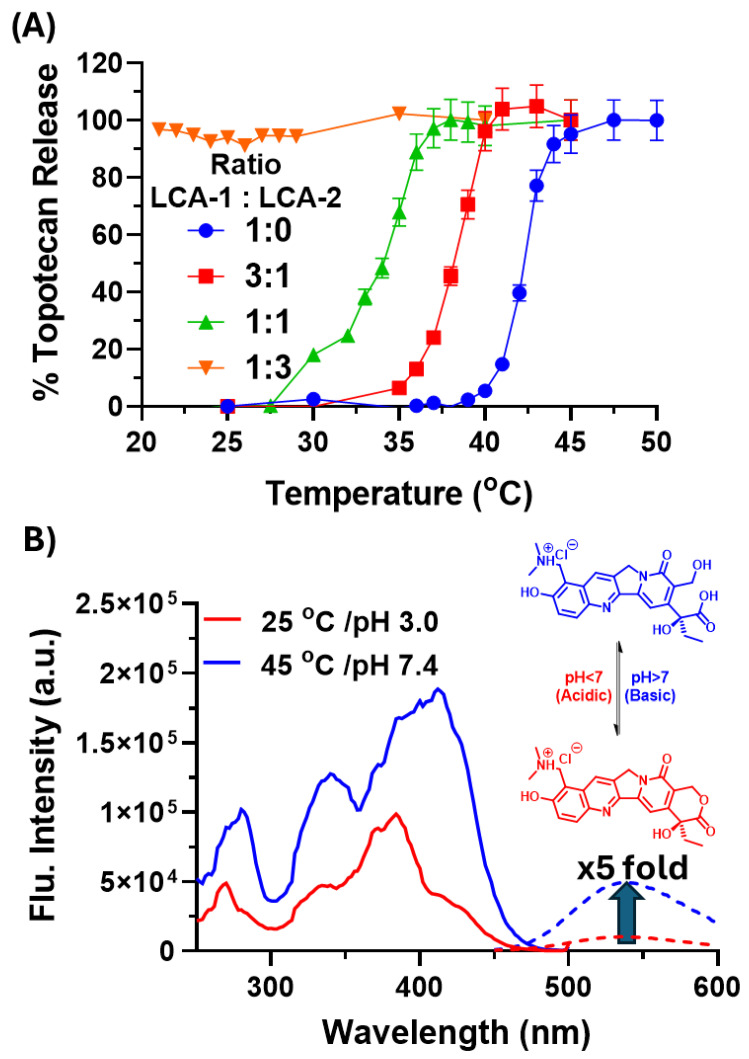
(**A**) **Thermally induced drug release** from the liposomes containing topotecan was assessed from an increase in fluorescence following the release of the encapsulated drug following incubation for 3 min at set temperatures (all values are mean average ± 1 SD; n = 3). (**B**) **Excitation (solid) and emission (dashed) spectra of topotecan** at pH 3 (red) and pH 7.4 (blue).

Previous work examined the topotecan release from liposomes via an increase in fluorescence intensity between encapsulated and released states [29,30]. This is due to topotecan having a UV/visible absorbance profile that is pH-sensitive and undergoes a red shift from an A_max_ of 385 nm at pH ~ 6 to a value of 414 nm at pH > 7. Since the central core of the liposomes is acidic, the fluorescence emission is greatly reduced for encapsulated topotecan when excited at 414 nm. Once the drug is released into a neutral or basic solution, the shift in the absorbance band is accompanied by a large increase in fluorescence emission at 530 nm. Therefore, by measuring changes in fluorescence intensity as a function of time at various temperatures, the extent of drug release could be observed in real time. To determine if the previously reported increase in fluorescence intensity was conserved in this formulation, the emission and excitation spectra were recorded at 414/530 nm at room temperature (encapsulated drug—pH 3) and then following heating at 45 °C for 5 min (released drug—pH 7.4) (Figure 3B).

The results showed a 5-fold increase in the fluorescence intensity (Figure 3B) between the room-temperature and heated (45 °C) samples. The liposome formulations with varying ratios of **LCA-1**:**LCA-2** (1:0, 3:1, 1:1, 1:3, and 0:1) were diluted 100-fold, then heated at temperatures from 20 to 50 °C for 3 min. Drug release was then determined by the aforementioned increase in fluorescence intensity against the 20 °C sample.

The drug-release curves of the **LCA-1**:**LCA-2** ratios (1:0, 3:1, and 1:1) showed a significant decrease in the temperature required to induce 50% drug release, shifting from 42.3 to 38.2 and 34.1 °C, respectively (Figure 3A). Alongside this, the release curve for **LCA-1**:**LCA-2** (1:3) shows that the encapsulated topotecan is released at room temperature, as no change in fluorescence intensity was observed upon heating, with the release curve for **LCA-1**:**LCA-2** (0:1) not able to be determined due all the topotecan being removed in the PD10 purification. This indicates that the higher the substitution of **LCA-1** with **LCA-2**, the more permeable the membrane becomes at lower temperatures. This is due to the shorter alkyl chain in **LCA-2** (C_16_) destabilising the membrane structure of the liposomes. This destabilisation reduces the temperature required for the gel-to-liquid-crystalline phase transition, allowing the topotecan to be released at lower temperatures. This highlights the potential of **LCA-1** and **LCA-2** combinations to fine-tune the thermal drug release.

### 2.5. Relaxometry of the Various **LCA-1**:**LCA-2** Liposomes

**Figure 4:**
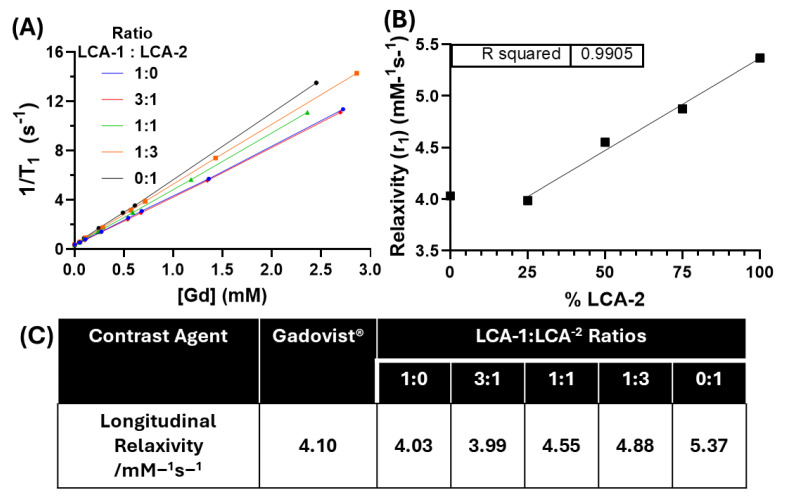
**T_1_ relaxivity measurements** of MRI contrast liposomes. (**A**) The relaxivity of the formulations with varying ratios of **LCA-1**:**LCA-2**: 1:0 (blue), 3:1 (red), 1:1 (green), 1:3 (orange), and 0:1 (black). T_1_ values were determined using a 400 MHz (9.4 T) NMR at ambient temperature. The relaxivity (r_1_) was determined by linear regression fitting. (**B**) Relationship between relaxivity (r_1_) and LCA1 substitution shows highly conserved linearity via linear regression (R^2^ = 0.9905). (**C**) Tabulated relaxivity (r_1_) values of a clinically used contrast agent Gadovist^®^ (Bayer, Barmen, Germany) and the liposome formulations determined from the linear regressions in A [31].

In order to determine if MR contrast was affected by the varied **LCA-1**:**LCA-2** ratio, NMR relaxometry studies were undertaken. The longitudinal relaxation time (T_1_) was determined using a standard saturation recovery sequence on a 400 MHz spectrometer (9.4 T) at 25 °C. The final relaxivity values of the liposomes were determined by plotting 1/T_1_ against [Gd] (Figure 4A). The relaxivity of the free lipids could not be obtained, as the CMC (critical micelle concentration) did not allow for a stable suspension at a suitable concentration for relaxivity studies.

All liposomal formulations displayed relaxivity values greater than or equal to the clinically available contrast agent Gadovist^®^ (4.10 mM ^−1^ s^−1^) and similar gadolinium-labelled liposomes (4.0 mM^−1^ s^−1^) at 9.4 T (Figure 4C) [31,32]. There is a linear correlation between relaxivity and the % incorporation of **LCA-2** (r_1_) (R^2^ = 0.9905) (Figure 4B). We hypothesise that this linear correlation between relaxivity and **LCA-2** incorporation is due to the increase in the membrane permeability to water. The **LCA-1**:**LCA-2** liposomes with ratios of 1:0 and 3:1 show no observed difference in relaxivity; we hypothesise that this is due to both formulations having similar water permeability at 25 °C. This hypothesis concurs with the information in both the DSC and topotecan release experiments (Figure 2A and Figure 3A). In addition, due to the incorporation of MSPC (lysolipid) into the liposomal formulation, there is potential pore formation in the membranes of the highly substituted **LCA-2** formulations (1:3, 0:1, (**LCA-1**:**LCA-2**)). This pore formation at lower temperatures would greatly increase water permeability across the lipid membrane, increasing water access to the intraliposomal facing contrast lipids (~50%) and resulting in the observed linear increase in relaxivity. It is also worth noting that the magnetic field strength could also affect this observed relationship, as highlighted in a previous study where liposomes composed of Gd-DOTA lipid amphiphiles showed that relaxivity was highly dependent on the magnetic field strength [33]. This is in line with previous literature that also states that the magnetic field strength and temperature strongly influence the contrast ability of such paramagnetic MRI contrast agents [34].

The increase in relaxivity of membrane-bound MR contrast agents, due to an increase in membrane permeability, could be a key tool in thermosensitive liposomes as a method of monitoring drug release in MRI-guided drug delivery. While this approach has been studied using encapsulated small-molecule contrast agents, this approach allowed only a very short window in which to image the contrast agent due to rapid release and diffusion, limiting the potential of encapsulated contrast agents to be used in the clinic for monitoring drug release. A membrane-bound alternative would be preferential, as this would lengthen the potential imaging window, resulting in less specialised MRI sequences being required for imaging [35,36,37,38]. Therefore, this study highlights the need for a library of imaging lipids that vary in tail length as a method to tune both the imaging and thermal release properties of liposomal formulations. This is increasingly important due to the fact that different drugs require different liposomal formulations for optimal drug encapsulation and release. So, by developing a library of imaging lipids with known effects on phase behaviour, a large step towards optimisation could be achieved computationally [39].

More drugs can be tested in the future to assess if the molecular weight and LogP have a significant effect on the finetuning ability of the membrane lipids or if it is conserved over multiple drug types. Future studies will focus on the development of these formulations for monitoring treatments of tumours.

## 3. Materials and Methods

Commercially available reagents and solvents were used without further purification. 1,2-Dipalmitoyl-sn-glycero-3-phosphocholine (DPPC; 16:0 PC), 1,2-distearoyl-sn-glycero-3- phosphocholine (DSPC; 18:0 PC), 1-stearoyl-sn-glycero-3-phosphocholine (MSPC; 18:0 Lyso PC), and (ω-methoxy-polyethylene glycol 2000)-N-carboxy-1,2-distearoyl-sn-glycero-3-phosphoethanolamine (PEG2000-DSPE) were purchased from Sigma-Aldrich (St. Louis, MO, USA) or Avanti Polar Lipids (Alabaster, AL, USA). XenoLight750-NHS-ester from Perkin Elmer (Waltham, MA, USA). All other materials were purchased from Sigma-Aldrich and were of analytical grade. Other lipids were synthesised as described below.

^1^H (400 MHz) NMR spectra were recorded on a Bruker Advance 400 spectrometer (Bruker, Billerica, MA, USA) using residual chloroform or dichloromethane as internal standards. Results are reported as chemical shifts in ppm from TMS, with peaks described as s = singlet, br = broad singlet, d = doublet, t = triplet, q = quartet, m = multiplet, and coupling constants J given in hertz (Hz). Mass spectroscopy was carried out on a Thermo LCQ DECA XP (Thermo Fisher Scientific, Waltham, MA, USA) or Agilent HP1100 MSD spectrometer (Agilent Technologies, Andover, MA, USA), depending on availability. UV spectroscopy and fluorescence measurements were carried out on an Infinite 200 Pro, Tecan, plate reader (Tecan Group Ltd., Männedorf, Switzerland). DLS and ζ-potential measurements were carried out on a Nanoseries Nano ZS (Malvern Instruments, Worcestershire, UK). DSC measurements were carried out using a Nano DSC (TA Instruments, New Castle, DE, USA). Thin Layer Chromatography (TLC) was carried out on F254 silica gel 60 plates, with spots visualised using UV illumination or KMnO_4_ staining and developed with a heat gun. Flash column chromatography was performed on 40–63 µm silica gel. MRI studies were performed using a quadrature volume RF coil with an inside diameter of 33 mm (Rapid Biomedical, Rimpar, Germany) on a 9.4 T Bruker MRI scanner (Bruker, Billerica, MA, USA).

### 3.1. Gadolinium MRI Contrast Lipid Synthesis

**Tri-tert-butyl 2,2′,2″-(1,4,7,10-tetraazacyclododecane-1,4,7-triyl)-triacetate (2).**

Cyclen (**1**) (2.00 g, 11.2 mmol) and NaHCO_3_ (3.02 g, 35.8 mmol) were suspended in MeCN (200 mL). To this, a mixture of *t*-butyl bromoacetate (5.32 mL, 35.8 mmol) and MeCN (95 mL) was added dropwise over 30 min. The reaction was then left to stir for 72 h. The inorganic salts were removed via filtration, and the filtrate was reduced in vacuo. The resulting residue was suspended in toluene (20 mL) and left overnight to precipitate. The precipitate was collected via filtration and washed with ice-cold toluene (10 mL) to yield the product (3.94 g, 7.4 mmol, 66%); ^1^H-NMR (400 MHz, CDCl_3_): δ_H_ (ppm) 10.05 (s, 1 H), 3.40 (s, 4 H), 3.31 (s, 2 H), 3.12 (s, 4 H), 2.94 (s, 8 H), 2.90 (s, 4 H), 1.48 (s, 18 H), 1.47 (s, 9 H); ESI-MS Calcd. for C_26_H_50_CoN_4_O_6_ [M]^+^: 514.4 a.m.u. Found [M + H^+^]^+^: 515.4 a.m.u.

**Tri-tert-butyl 2,2′,2″-(10-(2-(benzyloxy)-2-oxoethyl)-1,4,7,10-tetraazacyclododecane-1,4,7-triyl)-triacetate (3).**

Compound **2** (3.94 g, 7.66 mmol) and K_2_CO_3_ (2.11 g, 15.3 mmol) were dissolved in MeCN (100 mL). To this, a solution of benzyl bromoacetate (2.42 mL, 3.51 g, 15.3 mmol) was added dropwise over 30 min. The reaction was refluxed for 48 h. The inorganic salts were removed via filtration. The crude product was further purified via silica gel column chromatography (DCM: MeOH 9:1). (3.66 g, 72%, 5.32 mmol) ^1^H-NMR (400 MHz, CDCl_3_): δ_H_ (ppm) δ 7.34–7.25 (m, 5 H), 5.16–5.04 (m, 2 H), 3.36–2.01 (m, 24 H), 1.43 (s, 9 H), 1.39 (s, 18 H); ESI-MS Calcd. for C_35_H_58_N_4_O_8_ [M]^+^: 662.87 a.m.u. Found [M + Na^+^]^+^: 685.7 a.m.u.

**2-(4,7,10-tris(2-(tert-butoxy)-2-oxoethyl)-1,4,7,10-tetraazacyclododecan-1-yl)-acetic acid (4).**

Compound **3** (3.20 g, 4.8 mmol) was dissolved in MeOH (30 mL). To this, Pd(OH)_2_/C (320 mg, 10% *w*/*w*) was added and agitated under a hydrogen atmosphere (50 psi) using a hydrogenator. The catalyst was removed via filtration, and the filtrate was reduced to yield the product (2.6 g, 4.5 mmol, 94%); ^1^H-NMR (400 MHz, CDCl_3_): δ_H_ (ppm) 4.07 (s, 2 H), 3.81–2.09 (m, 22 H), 1.56–1.36 (m, 27 H); ESI-MS Calcd. for C_28_H_52_N_4_O_8_ [M]^+^: 572.4 a.m.u. Found [M + Na^+^]^+^: 595.4 a.m.u.

**Tert-butyl (2-(dioctadecylamino)-2-oxoethyl)carbamate (6).**

Compound **5** (1.788 g, 3.43 mmol), OH.Gly.BOC (600 mg, 3.43 mmol), and HBTU (1.556 g, 4.1 mmol) were dissolved in dry DCM (50 mL). To this, DIPEA (1.65 g, 2.23 mL, 10.28 mmol) was added, and the reaction was stirred at rt. for 24 h. The volatiles were removed in vacuo. The resulting residue was dissolved in DCM (50 mL) and washed with water (3 × 100 mL), 7% citric acid (3 × 100 mL), and brine (3 × 100 mL). The organic layers were combined and dried over MgSO_4_ to yield the product (2.09 g, 3.1 mmol, 90%); ^1^H-NMR (400 MHz, CDCl_3_): δ_H_ (ppm) 5.50 (s, 1 H), 3.87 (d, *J* = 3.9 Hz, 2 H), 3.27–3.21 (t, *J* = 7.5, 2 H), 3.13–3.03 (t, *J* = 7.5, 2 H), 1.46 (s, 4 H), 1.38 (s, 9 H), 1.19 (s, 60 H), 0.81 (t, *J* = 6.8 Hz, 6 H); ESI-MS Calcd. for [M]^+^: 679.2 a.m.u. Found [M + H]^+^: 680.0 a.m.u.

**2-amino-N,N-dioctadecylacetamide (7).**

Compound **6** (2.0 g, 3.1 mmol) was dissolved in DCM (7 mL). To this, TFA (3 mL) was added and stirred overnight. The volatiles were removed in vacuo. The crude residue was dissolved in minimum DCM before being added to diethyl ether (150 mL). The cloudy suspension was cooled at −20 °C for 2 h. The resulting ppt. was collected via filtration to yield the product as a TFA salt (2.11 g, 3.1 mmol, 97%); ^1^H-NMR (400 MHz, CDCl_3_): δ_H_ 8.47 (s, 2 H), 3.86 (s, 2 H), 3.31 (t, *J* = 7.5, 2 H), 3.13 (t, *J* = 7.5, 2 H), 1.60–1.45 (m, 4 H), 1.28 (br, 60 H), 0.90 (t, *J* = 6.7 Hz, 6 H); ESI-MS Calcd. for C_38_H_78_N_2_O [M]^+^: 578.6 a.m.u. Found [M + H^+^]^+^: 579.3 a.m.u.

**Tri-tert-butyl-2,2′,2″-(10-(2-((2-(dioctadecylamino)-2-oxoethyl)amino)-2-oxoethyl)-1,4,7,10-tetraazacyclododecane-1,4,7-triyl)-triacetate (8).**

Compound **7** (850 mg, 1.47 mmol), **4** (880 mg, 1.54 mmol), and HBTU (667 mg, 1.76 mmol) were dissolved in dry DCM (50 mL). To this, DIPEA (765 µL, 567 mg, 4.4 mmol) was added, and the resulting mixture was stirred at rt. for 18 h under N_2(g)_. The volatiles were removed in vacuo. The resulting residue was dissolved in DCM (100 mL) and washed with water (3 × 200 mL) and brine (3 × 200 mL). The organic layers were combined and dried over MgSO_4_. The product was then purified via silica gel column chromatography [MeOH: DCM (2:98)] to yield the product (754 mg, 0.66 mmol, 45%); ^1^H-NMR (400 MHz, CDCl_3_): δ_H_ 6.62 (s, 1 H), 4.00 (s, 2 H), 3.46–2.04 (m, 28 H), 1.56 (s, 4 H), 1.47 (d, *J* = 6.0 Hz, 27 H), 1.27 (br, 60 H), 0.90 (t, *J* = 6.7 Hz, 6 H); ESI-MS Calcd. for C_66_H_128_N_6_O_8_[M]^+^: 1133.0 a.m.u. Found [M + Na^+^]^+^: 1154.6 a.m.u.

**2,2′,2″-(10-(2-((2-(dioctadecylamino)-2-oxoethyl)amino)-2-oxoethyl)-1,4,7,10-tetraazacyclododecane-1,4,7-triyl)-triacetic acid (9).**

Compound **8** (536 mg, 0.47 mmol) was suspended in dioxane/HCL (4M) (5 mL) and refluxed for 24 h. The volatiles were removed s, and the crude product was purified via silica gel column chromatography using a gradient of [(DCM:MeOH: NH_3_ (78:20:2)): DCM (2:8 -> 9:1)] to yield the product (403 mg, 0.42 mmol, 89%); ^1^H-NMR (400 MHz, CDCl_3_): δ_H_ 7.54 (s, 1 H), 4.22–2.13 (m, 30 H), 1.53 (d, *J* = 28.7 Hz, 4 H), 1.28 (s, 60 H), 0.90 (t, *J* = 6.7 Hz, 6 H); ESI-MS Calcd. for C_54_H_104_N_6_O_8_ [M]^+^: 964.8 a.m.u. Found [M + Na^+^]^+^: 987.7 a.m.u.

**LCA-1 (10, [Gd]-2,2′,2″-(10-(2-((2-(dioctadecylamino)-2-oxoethyl)amino)-2-oxoethyl)-1,4,7,10-tetraazacyclododecane-1,4,7-triyl)-triacetic acid).**

Compound **9** (1.1 g, 1.14 mmol) mmol) was suspended in water (50 mL). GdCl_3_.6H_2_O (464 mg, 1.25 mmol) was dissolved in water (5 mL) and added dropwise over 30 min. The reaction was refluxed for 48 h, with the pH maintained at pH 6. The suspension was washed using water (3 × 50 mL) via centrifugation (3000× *g* rpm, 10 min), and the supernatant was removed. The product was dried in vacuo. The residue was dissolved in CHCl_3_ and centrifuged to remove trace inorganic salts before the supernatant was removed and dried in vacuo to yield the product as a white powder (880 mg, 0.79 mmol, 70%); ESI-MS Calcd. for C_54_H_101_GdN_6_O_8_ [M]^+^: 1119.7 a.m.u. Found [M + H^+^]^+^: 1120.1 a.m.u.

**Dihexadecylamine (12).**

Hexadeylamine (**11**) (3.78 g, 15.72 mmol), 1-bromohexadecane (4 g, 13.08 mmol), and K_2_CO_3_ (4.52 g, 32.76 mmol) were refluxed in MeCN (100 mL) for 120 h. The inorganic salts were removed via filtration, and the filtrate was reduced in vacuo. The residue was dissolved in CHCl_3_ and washed with aqueous (sat)Na_2_CO_3_ and water (3 × 100 mL) before drying over MgSO_4_. The solvent was removed in vacuo, and the crude product was recrystallised from hexane to yield the product in white powder (3.8398 g, 8.21 mmol, 63% yield). ^1^H-NMR (400 MHz, CDCl_3_); δ_H_ (ppm) 2.51 (t, *J* = 7.3 Hz, 4 H), 1.60–1.32 (m, 4 H), 1.20 (d, *J* = 8.9 Hz, 52 H), 0.81 (t, *J* = 6.8 Hz, 6 H); ESI-MS Calcd. for C_32_H_67_N [M]^+^: 465.5 a.m.u. Found [M + H^+^]^+^: 466.4 a.m.u.

**Tert-butyl (2-(dihexadecylamino)-2-oxoethyl)-carbamate HSA.G.BOC (13).**

Compound (**12**) (1.2 g, 2.57 mmol), HO.Gly.NH.BOC (451 mg, 2.57 mmol), and HBTU (1.17 g, 3.1 mmol) were dissolved in dry DCM (40 mL). To this, DIPEA (1.46 mL, 1.085 g, 8.4 mmol) was added, and the reaction was stirred under N_2(g)_ for 18 h. The volatiles were removed in vacuo. The residue was dissolved in DCM (50 mL) and washed with water (3 × 100 mL), brine (3 × 100 mL), and 7% *w*/*v* citric acid (3 × 100 mL). The organic layers were combined and dried over MgSO_4_ to yield the product (1.2 g, 1.9 mmol, 75%); ^1^H-NMR (400 MHz, CDCl_3_): δ_H_ 5.55 (s, 1 H), 3.93 (d, *J* = 3.4 Hz, 2 H), 3.34–3.27 (m, 2 H), 3.16–3.09 (m, 2 H), 1.51 (s, 4 H), 1.44 (s, 9 H), 1.25 (s, 52 H), 0.87 (t, *J* = 6).

**2-amino-N,N-dihexadecylacetamide (14).**

Compound **13** (1.2 g, 1.9 mmol) was dissolved in DCM (7 mL). To this, TFA (3 mL) was added and stirred at rt for 6 h. The volatiles were removed in vacuo. The residue was dissolved in minimum DCM and added to diethyl ether (100 mL). The solution was cooled to −20 °C for 2 h. The resulting precipitate was collected and washed with ice-cold ether to yield the product. (975 mg, 1.9 mmol, 97%); ^1^H-NMR (400 MHz, CDCl_3_): δ_H_ 8.45 (s, 2 H), 3.86 (s, 2 H), 3.32 (s, 2 H), 3.14 (s, 2 H), 1.48 (s, 4 H), 1.28 (s, 52 H), 0.90 (t, *J* = 6.7 Hz, 6 H); ESI-MS Calcd. for C_34_H_70_N_2_O [M]^+^: 522.5 a.m.u. Found [M + H^+^]^+^: 523.4 a.m.u.

**Tri-tert-butyl-2,2′,2″-(10-(2-((2-(dihexadecylamino)-2-oxoethyl)amino)-2-oxoethyl)-1,4,7,10-tetraazacyclododecane-1,4,7-triyl)-triacetate (15).**

Compound 14 (975 mg, 1.86 mmol), 4 (1.07 g, 1.86 mmol), and HBTU (848 mg, 2.24 mmol) were dissolved in dry DCM (40 mL). To this, DIPEA (9.75 mL, 723 mg, 5.6 mmol) was added, and the resulting mixture was stirred at rt. for 18 h under N_2(g)_. The volatiles were removed in vacuo. The resulting residue was dissolved in DCM (100 mL) and washed with water (3 × 100 mL) and brine (3 × 100 mL). The organic layers were combined and dried over MgSO_4_ to yield the product (1.41 g, 1.3 mmol, 70%); ^1^H-NMR (400 MHz, CDCl_3_): δ_H_ 6.66 (s, 1 H), 3.99 (s, 2 H), 3.49–3.28 (m, 4 H), 3.20–3.10 (m, 4 H), 3.03–1.77 (m, 20 H), 1.56 (s, 4 H), 1.46 (d, *J* = 6.3 Hz, 27 H), 1.26 (s, 52 H), 0.88 (t, *J* = 6.6 Hz, 6 H); ESI-MS Calcd. for C_62_H_120_N_6_O_8_ [M]^+^: 1076.9 a.m.u. Found [M + Na^+^]^+^: 1100.0 a.m.u.

**2,2′,2″-(10-(2-((2-(dihexadecylamino)-2-oxoethyl)amino)-2-oxoethyl)-1,4,7,10-tetraazacyclododecane-1,4,7-triyl)-triacetic (16).**

Compound 15 (1.0 g, 0.93 mmol) was suspended in HCl (4M): dioxane (5 mL) and refluxed for 4 h. The volatiles were removed in vacuo. The crude product was purified via silica gel column chromatography [DCM:(DCM:MeOH: NH_3_(78:20:2)] (640 mg, 0.70 mmol, 75%); ^1^H-NMR (400 MHz, CDCl_3_): δ_H_ 4.23–2.14 (m, 34 H), 1.51 (s, 4 H), 1.28 (s, 52 H), 0.90 (t, *J* = 6.4 Hz, 6 H).; HR ESI-MS Calcd. for C_50_H_96_N_6_O_8_ [M]^+^: 908.7 a.m.u. Found [M]^+^: 909.8 a.m.u.

**LCA-2 (17, [Gd]-2,2′,2″-(10-(2-((2-(dihexadecylamino)-2-oxoethyl)amino)-2-oxoethyl)-1,4,7,10-tetraazacyclododecane-1,4,7-triyl)-triacetic).**

Compound 16 (640 mg, 0.7 mmol) was suspended in water (50 mL). GdCl_3_.6H_2_O (288 mg, 0.77 mmol) was dissolved in water (5 mL) and added dropwise over 30 min. The reaction was at reflux for 48 h, with the pH maintained at pH 6. The suspension was washed using water (3 × 50 mL) via centrifugation (3000× *g* rpm, 10 min), and the supernatant was removed. The product was dried in vacuo. The residue was dissolved in CHCl_3_ and centrifuged to remove trace inorganic salts before the supernatant was removed and dried in vacuo to yield the product as a white powder (600 mg, 0.56 mmol, 80%); ESI-MS Calcd. for C_50_H_93_GdN_6_O_8_ [M]^+^: 1063.6 a.m.u. Found [M]^+^: 1064.2 a.m.u.

### 3.2. Liposome Preparation

All lipids were stored in aliquots (20 mg/mL) in CHCl_3_. Liposomes were prepared with the following lipid formations: DPPC: MSPC: PEG_2000_-DSPE: LCA (55.3: 9.7: 5: 30 mol%) [40]. The different ratios of **LCA-1** and **LCA-2** were calculated with the total mol% equalling 30 mol% of the formulation (**1:0** = 30 mol % **LCA-1**; **3:1** = 22.5 mol % and **LCA-1**/7.5 mol% **LCA-2**; **1:1** = 15 mol% **LCA-1** and 15 mol% **LCA-2**; **1:3** = 7.5 mol% **LCA-1** and 22.5 mol% **LCA-2**; **0:1** = 30 mol% **LCA-2**). Lipid stocks were combined in a round-bottom flask in proportion to their respective mol% values (total mass of lipid 45 mg). The solvent was slowly evaporated in vacuo to ensure a thin and even film formation. Liposome formulations were hydrated in 300 mM ammonium sulphate (1.5 mL), topotecan (1 mg/mL), pH 3. The film was fragmented by alternately plunging into liquid nitrogen and then hot water (×10 times). The resulting suspension was sonicated at 60 °C for long enough to form a homogeneous opalescent solution. Free topotecan was removed using size exclusion column chromatography (PD-10) using 20 mM HEPES, 5% glucose, pH 7.4 buffer. Purified liposomes were stored at 4 °C until used.

### 3.3. DLS and ζ-Potential

DLS and ζ-potential measurements were carried out on diluted samples 1:100 *v*/*v*. Each DLS measurement included an equilibration time of 60 s before recording, which included 3 repetitions of 15 measurements. The reported size values were obtained by a distribution analysis of the data. Each ζ potential measurement included 5 repetitions (the number of scans was determined by the device depending on the consistency of the data).

### 3.4. Differential Scanning Calorimetry

Differential scanning calorimetry was used to assess the liposomes’ phase transition. Liposomes were diluted to ~1 mg/mL lipid into degassed 20 mM HEPES, 5% *w*/*v* glucose buffer, pH 7.4. Samples (600 µL) were then loaded into a Nano DSC (TA Instruments, New Castle, DE, USA), and 3 rounds of sequential heating/cooling (25–70 °C at 1 °C/min) were then carried out against a reference of the same buffer. Each scan sequence was also carried out in triplicate with new samples of liposomes.

### 3.5. Topotecan Release In Vitro

The triggered drug release of topotecan was assessed using fluorescence measurements. Studies were carried out with separate samples diluted 1:100 (*v*/*v*) in 20 mM HEPES buffer (pH 7.4), then incubated in a Thermocycler (25–45 °C; 3 min) before being cooled to ambient temperature. After transfer to a 96-well plate, the fluorescence (Ex 414 nm b.w. 9 nm; Em 530 nm b.w. 20 nm) emitted from individual samples was measured using a plate reader. The complete release was assessed after the liposomes were heated (5 min; 45 °C) to allow topotecan to be released in the external buffer.

### 3.6. Relaxivity (r_1_) Measurements

A series of dilutions were prepared: ×2, 4, 8, 10, 20, 50, and 100 in 20 mM HEPES and 5% glucose for the liposome formulations. In addition, all samples were made to contain a final concentration of 10% D_2_O. A 400 MHz Bruker Advance 400 NMR spectrometer (Bruker, Billerica, MA, USA) was used to obtain T_1_ relaxation times using standard saturation recovery method with recycle delays for 5 ms and delay times (Ti) of 2 ms to 3 s. T_1_ values were determined by exponentially fitting to the following equation:

(1) Mi=M0(1−e−TRT1)

Equation (1): Saturation recovery equation was used to determine T_1_ values. M_i_ = measured signal for a given T_R_, T_R_ = time to repeat, T_1_ = longitudinal relaxation time, M_0_ = baseline signal.

(2) Mi=M0(1−e−TET2).

Equation (2): Spin echo equation was used to determine the T_2_ values. M_i_ = measured signal for a given T_E_, T_E_ = Echo time, T_2_ = transverse relaxation time, and M_0_ = baseline signal.

The gadolinium concentration of the liposomes was determined using ICP-MS. Liposomes (10 µL) were digested in HNO_3_ (140 µL) and H_2_O_2_ (50 µL) overnight at 60 °C. The digested sample was diluted 10-fold with ultrapure water, and the gadolinium concentrations were analysed using ICP-MS (PerkinElmer NexION 350D). Longitudinal relaxivity (r_1_) was determine by linear regression of corrected T1 (T_1_ − T_0_) vs. [Gd] using following equation:

(3) r1=(1(T1)−1T0)[CA]

Equation (3): relaxivity equation used to obtain r_1_. 1/T_1_ = longitudinal relaxation rate, T_0_ = longitudinal relaxation rate of the buffer, [CA] = concentration of contrast agent (Gadolinium concentration in this case), and r_1_ = longitudinal relaxivity.

## 4. Conclusions

**LCA-1** and **LCA-2** were successfully synthesised via ESI-MS confirmation. The MR contrast lipids were incorporated into thermosensitive liposomal formulations at varying ratios of **LCA-1** (C_18_):**LCA-2** (C_16_), resulting in a linear decrease in the phase transition temperature (R^2^ = 0.9251). Additionally, the relaxivity of the liposomal formulations increased with increasing **LCA-2**, which we hypothesise is the result of an increase in membrane permeability to water. Overall, this study shows that the composition and combination of MRI lipids strongly affect the thermal drug release properties of the liposomes, and such lipids could be used for fine-tuning the lipid membrane thermosensitivity while maintaining clinically relevant relaxivity. In conclusion, based on our studies, we anticipate that combinations of **LCA-1** and **LCA-2** lipids may be used to monitor liposomal drug release with MRI in the clinic using clinically relevant magnetic field strengths.

## Data Availability

The data presented in this study are available upon request from the corresponding author.
